# Supplementary material for: COZOID: contact zone identifier for visual analysis of protein-protein interactions
Source: BMC Bioinformatics. 2018 Apr 6;19:125. doi: 10.1186/s12859-018-2113-6 (PMC5889581; doi:10.1186/s12859-018-2113-6)
Supplement: Supplementary file 2 — Software build. Executable binary file of the software tool. (ZIP 118,784 kb) [file 12859_2018_2113_MOESM2_ESM.zip › cozoid/java/docs/beansbinding-1.2.1-doc.zip!/org/jdesktop/el/impl/lang/package-summary.html]

org.jdesktop.el.impl.lang (Beans Binding)


---


|  |  |  |
| --- | --- | --- |
| **Overview** \| **Package** \| Class \| **Use** \| **Tree** \| **Deprecated** \| **Index** \| **Help** | |  |
| **PREV PACKAGE**   **NEXT PACKAGE** | **FRAMES**    **NO FRAMES**     **All Classes** |  |


---

## Package org.jdesktop.el.impl.lang

Customized version of EL for Beans Binding; not for general use.

**See:**
  
          **Description**

| **Class Summary** | |
| --- | --- |
| **ELArithmetic** | A helper class of Arithmetic defined by the EL Specification |
| **ELArithmetic.BigDecimalDelegate** |  |
| **ELArithmetic.BigIntegerDelegate** |  |
| **ELArithmetic.DoubleDelegate** |  |
| **ELArithmetic.LongDelegate** |  |
| **ELSupport** | A helper class that implements the EL Specification |
| **EvaluationContext** |  |
| **ExpressionBuilder** |  |
| **FunctionMapperFactory** |  |
| **FunctionMapperImpl** |  |
| **FunctionMapperImpl.Function** |  |
| **VariableMapperFactory** |  |
| **VariableMapperImpl** |  |

## Package org.jdesktop.el.impl.lang Description

Customized version of EL for Beans Binding; not for general use.
It is expected that we'll sync up with an official version of EL
in the future.

---


|  |  |  |
| --- | --- | --- |
| **Overview** \| **Package** \| Class \| **Use** \| **Tree** \| **Deprecated** \| **Index** \| **Help** | |  |
| **PREV PACKAGE**   **NEXT PACKAGE** | **FRAMES**    **NO FRAMES**     **All Classes** |  |


---
